# Effects of Empagliflozin on Progression of Chronic Kidney Disease: A Pre-Specified Secondary Analysis from the Empa-Kidney Trial

**DOI:** 10.1016/S2213-8587(23)00321-2

**Published:** 2023-12-04

**Authors:** The EMPA-KIDNEY Collaborative Group

**Keywords:** sodium glucose co-transporter-2 inhibitors, CKD, slope, progression

## Abstract

**Background:**

Sodium glucose co-transport 2 inhibitors (SGLT2i) reduce progression of chronic kidney disease (CKD) and the risk of cardiovascular morbidity and mortality in a wide range of patients. However, their effects on kidney disease progression in some patients with CKD are unclear because few clinical kidney outcomes occurred among such patients in the completed trials. In particular, some guidelines stratify their level of recommendation about who should be treated with SGLT2i based on diabetes status and albuminuria. Our aim was to assess the effects of empagliflozin on progression of CKD both overall and among specific types of participant in the EMPA-KIDNEY trial.

**Methods:**

We explored the effects of empaglifozin 10 mg once daily versus placebo on the annualised rate of change in estimated glomerular filtration rate (“eGFR slope”), a tertiary outcome in the EMPA-KIDNEY trial. We studied the acute and chronic slopes (from randomization to 2 months, and from 2 months onwards respectively) separately, using shared parameter models to estimate the latter. EMPA-KIDNEY is registered at clinicaltrials.gov (NCT03594110).

**Findings:**

Overall, allocation to empaglifozin caused an acute 2.12 (95% CI 1.83 to 2.41) mL/min/1.73m^2^ reduction in eGFR, equivalent to a 6% (5–6%) dip in the first 2 months. After this, it halved the chronic slope from -2.75 to -1.37 mL/min/1.73m^2^/year (relative difference 50%, 95% CI 42–58%). The absolute and relative benefits of empagliflozin on the magnitude of the chronic slope varied significantly depending on diabetes status and baseline levels of eGFR and uACR. In particular, the absolute difference in chronic slopes was lower in those with lower baseline uACR, but because this group progressed more slowly than those with higher uACR, this translated to a larger relative difference in chronic slopes in this group (76% [32–120%] reduction in the chronic slope among those with baseline uACR <30mg/g compared with a 28% [19–38%] reduction for those with baseline uACR ≥2000 mg/g; p for trend 0.0001).

**Interpretation:**

Empagliflozin slowed the rate of progression of CKD among all types of participant in the EMPA-KIDNEY trial, including those with little albuminuria. Albuminuria alone should not be used to determine whether to treat with an SGLT2i.

**Funding:**

EMPA-KIDNEY funded by a grant to the University of Oxford from Boehringer Ingelheim and Eli Lilly.

## Introduction

Chronic kidney disease (CKD) is common and associated with reduced quality of life and increased risks of kidney failure (which is fatal without costly kidney replacement therapy), cardiovascular disease and mortality.^1–3^ Trials in CKD populations have traditionally used dichotomous composite clinical outcomes which combine kidney failure (an infrequent outcome) with a proportional reduction in kidney function (as measured by change in estimated glomerular filtration rate [eGFR]) from baseline in excess of a particular threshold, usually 40% to 57%.^4^ The latter component is now accepted by regulatory authorities as a valid surrogate outcome for kidney failure itself in randomized trials.^4,5^ Like all dichotomous outcomes, the majority of such outcomes during the trial follow-up period occurs in patients at highest risk, unlike in the general population where the majority of events occur during the lifetime of patients at moderate risk because of the much larger number of such patients.^6^ Therefore analyses based on these outcomes have less statistical sensitivity for determining the effects of treatment among groups of patients who are at lower risk of kidney failure (including those with better preserved kidney function) but from whom ultimately come the majority of patients with kidney failure in the general population. There is therefore interest in examining the annualised rate of decline of kidney function (“eGFR slope”) because this parameter can be calculated for all participants, and so eGFR slopes have improved statistical sensitivity when comparing the effect of an intervention in different types of patient. It may also be considered as a valid surrogate of CKD progression *per se* and be used as a primary outcome in trials.^7,8^ Importantly, the effect of a treatment on eGFR slope is not necessarily homogeneous over time; many renoprotective treatments including renin-angiotensin system (RAS) inhibitors, sodium-glucose co-transporter-2 inhibitors (SGLT2i) and finerenone, cause an early ‘acute’ negative effect on slope (referred to as an acute dip), followed by a long-term (or ‘chronic’) reduction in eGFR slope. It is therefore important to understand the effects of new treatments on these two components of the ‘total’ slope.

Randomized trials of SGLT2i have consistently shown that this class of treatment reduces the risk of progression of kidney disease (measured with dichotomous outcomes) in patients with CKD with or without diabetes, irrespective of underlying primary kidney disease.^9–12^ Secondary analyses of two previous trials of SGLT2i in CKD populations found some evidence that the effects of SGLT2i on eGFR slope varied in different types of patient, but because the trials only recruited patients with significant albuminuria, they were limited in their ability to explore whether this effect varied according to baseline albuminuria as well as other clinical characteristics.^13,14^ Here we present the effects of empagliflozin versus placebo on eGFR slope from the EMPA-KIDNEY trial which recruited a uniquely broad range of patients with CKD at risk of progression, including those with minimal albuminuria, low eGFR, and with and without diabetes.

## Methods

Details of EMPA-KIDNEY’s rationale, design, protocol, pre-specified data analysis plan, and main results have been reported previously.^11,15,16^ The trial was conducted at 241 centres in 8 countries. Regulatory authorities and ethics committees for each centre approved the trial. Adults with a race-adjusted CKD-EPI^17^ eGFR of ≥20 <45 ml/min/1.73m^2^ (irrespective of level of albuminuria); or an eGFR of ≥45 <90 ml/min1.73m^2^ with a urinary albumin-to-creatinine ratio (uACR) ≥200 mg/g at the screening visit were eligible provided they were prescribed a clinically appropriate dose of single-agent RAS-inhibitor, where indicated and tolerated. Patients with or without diabetes mellitus were eligible and polycystic kidney disease was the only excluded primary kidney disease. Those receiving at least 45mg prednisolone daily (or equivalent) or had received intravenous immunosuppression in the last 3 months were excluded.

All eligible and consenting participants entered a pre-randomization run-in phase and were provided with a 15-week supply of once daily placebo tablets. During this time, local investigators reviewed screening data, assessed current RAS-inhibitor use, and approved potential participants for later randomization. Participant-reported primary kidney disease was confirmed by local lead investigators. Throughout the trial, clinical responsibility for participants remained with their local doctors. After completing the run-in, willing and eligible participants had central samples of blood and urine collected for central analysis and long-term storage, and were randomly allocated to receive empagliflozin (10 mg once daily) or matching placebo.^18^ At follow-up visits, participants provided information on renal status (i.e. any dialysis treatment or receipt of a kidney transplant), adherence to study treatment (with reasons for stopping) and details of concomitant medication. They were also asked in a structured interview about any serious adverse events (and protocol-specified non-serious adverse events), underwent clinical measurements of blood pressure and weight, and had blood collected for local safety assessment of creatinine, liver function and potassium. Blood samples and, at selected visits, urine samples were sent to the central laboratory for efficacy analyses and archiving. Surviving participants in the UK were asked to provide a blood sample for local laboratory analysis of creatinine about 4 weeks after their final follow-up visit in order to assess the effect of discontinuing empagliflozin on eGFR.

### Outcomes

Annual rate of change in eGFR calculated separately for the period from baseline to the final follow-up visit (i.e. “total slope”) and for the period from 2 months to the final follow-up visit (i.e. “chronic slope”) were tertiary outcomes in the original protocol, with exploratory analyses of these outcomes pre-specified. Acute dips in eGFR were calculated as the difference in eGFR between baseline and the 2 month follow-up visit.

### Statistical analyses

Unless stated otherwise, all analyses were performed according to the intention-to-treat principle. Effects of empagliflozin on annual rate of change in eGFR were assessed using pre-specified shared parameter models,^19,20^ which were used to calculate the chronic eGFR slope. For subgroup analyses, absolute differences in chronic slopes were calculated and from these relative differences were calculated by dividing the absolute effect and its 95% confidence interval (CI) by the mean slope in the placebo arm. Pre-specified subgroup categories for eGFR and uACR were <30, ≥30<45, ≥45 mL/min/1.73m^2^ and <30, ≥30≤300, >300 mg/g respectively. The lowest eGFR and highest uACR categories were further subdivided to give post-hoc expanded subgroups (eGFR <20, ≥20<30, ≥30<45, ≥45 mL/min/1.73m^2^ and <30, ≥30≤300, >300<1000, ≥1000<2000, ≥2000 mg/g), and the pre-specified uACR subgroup was also divided by diabetes. In keeping with the pre-specified analyses of the chronic slopes, effects of empagliflozin on acute dips were estimated using linear regression models adjusted for baseline variables specified in the minimisation algorithm (age, sex, prior diabetes, eGFR, uACR, and region). Subgroup specific effects were estimated through the inclusion of treatment by subgroup interaction terms. Relative differences in acute dips as a percentage of mean baseline eGFR were also calculated. Standard tests for heterogeneity or trend across subgroups were performed for relative differences in eGFR slopes and acute dips.

Sensitivity analyses for annual rate of change in eGFR included the addition of interactions with other key subgroups (to standardize the distribution of other characteristics across subgroups), as well as restricting analyses to on-treatment eGFR measurements and using eGFR measurements based on local creatinine values. In the surviving UK participants with a 4-week post-final follow-up blood sample, the effect of stopping study treatment on mean eGFR (after taking account of any differences in eGFR at final follow-up) was estimated using linear regression models adjusted for age, sex, prior diabetes, and uACR as specified in the minimisation algorithm and eGFR at the final follow-up visit. Effects of empagliflozin on albuminuria used a pre-specified mixed model repeated measures (MMRM) approach.^11^ The normality of residuals assumption was examined for each linear regression model and MMRM through the inspection of histograms and Q-Q plots. The assumption of homoscedasticity was assessed through visual inspection of a plot of fitted values against the residuals. No violations of the assumptions underlying the linear regression models or MMRM were identified.

The proportion of treatment effect for the primary composite outcome of kidney disease progression or cardiovascular death explained by on-study uACR, systolic blood pressure (SBP), diastolic blood pressure (DBP) and glycated hemoglobin (HbA1c) was estimated using the landmark method,^21,22^ adjusting the pre-specified Cox regression model for 2 month values of the biomarkers. Bias-corrected and accelerated bootstrap intervals with 10,000 replications were used to construct the 95% CIs. Time-to-event analyses defined time at risk as originating/starting from randomization, and finishing at the date of event of interest, final follow-up or censoring at the earliest of death, loss to follow-up or withdrawal of consent. Assessment of the proportional hazards assumption (by testing the significance of an interaction between treatment allocation and log[survival time]) found no evidence against proportionality for any of the time to event outcomes. The landmark method was also used to estimate the proportion of the treatment effect on chronic slope explained by the same on-study biomarkers. Changes in Wald *χ*^2^ statistics are also presented to quantify the reduction in the strength of the association between treatment allocation and outcomes after adjustment for 2 month biomarkers.

Further statistical details are provided in the previously published data analysis plan and primary report.^11^ Analyses used SAS software, version 9.4 (SAS Institute, Cary NY, USA) and R v4.3.0.

### Role of the funder

The main trial funder (Boehringer Ingelheim) has minority representation on the trial Steering Committee which provided oversight of trial design, data collection and interpretation. The first and senior authors are responsible for the analyses performed by the University of Oxford - where the original full database is held – and take responsibility for manuscript and the decision to submit.

## Results

### Baseline characteristics

Between May 2019 and April 2021, 6609 participants were randomized and then followed for a median of 2·0 years. Prespecified subgroups of eGFR included 2282 (35%), 2928 (44%) and 1399 (21%) with eGFR <30, ≥30 to <45 and ≥45 mL/min/1.73m^2^ respectively. Prespecified subgroups of uACR included 1328 (20%), 1864 (28%) and 3417 (52%) with uACR <30, ≥30 to ≤300 and >300 mg/g respectively (Table 1). Participants with lower eGFR were older and more likely to have diabetes. uACR was highest among participants with eGFR ≥45 mL/min/1.73m^2^, due to the requirement for them to have a uACR ≥200 mg/g at screening to be eligible. Those with higher uACR were younger, less likely to have diabetes and had a higher mean eGFR (Table 1). Baseline characteristics for the expanded eGFR and uACR categories are given in Webtable 1.

### Effect of empagliflozin on acute changes in eGFR and albuminuria

**Figure 1:**
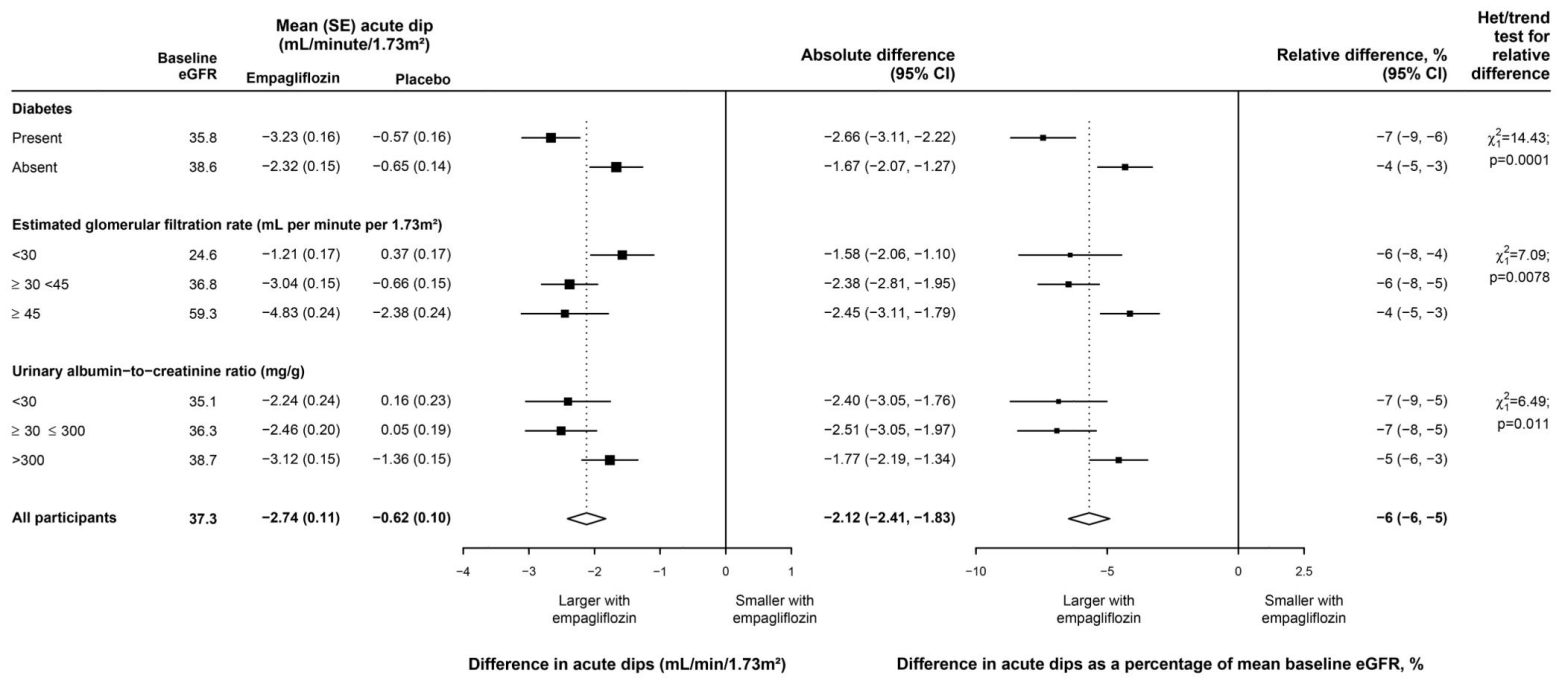
Effect of allocation to empagliflozin on acute changes in estimated glomerular filtration rate, by key subgroups P values for test of heterogeneity between absolute differences for patients with and without diabetes and tests for trend in absolute differences across eGFR and UACR categories are 0.0010, 0.016 and 0.050 respectively.

Between randomization and the 2 month follow-up visit, the placebo-adjusted ‘acute dip’ in eGFR with empagliflozin was 2.12 (95% CI 1.83–2.41) mL/min/1.73m^2^, or, in relative terms, 6% (5–6%). The relative effects varied significantly across the key subgroups (Figure 1) but were generally similar across other pre-specified subgroups with statistical evidence for some effect modification by age, body mass index, HbA1c and use of lipid lowering medication (Webfigure 1). In the surviving UK participants with a 4-week post-final follow-up blood sample, this acute dip reversed when study treatment was discontinued, with mean eGFR at 4 weeks post-final follow-up being of 29.8 mL/min/1.73m^2^ in the empagliflozin group and 27.9 mL/min/1.73m^2^ in the placebo group (difference 1.87 [95% CI 1.23–2.52] mL/min/1.73m^2^) (after accounting for any differences at final follow-up; Webtable 2), with similar differences observed in subgroup analyses by baseline eGFR and uACR categories (Webtable 3).

The geometric mean study average uACR was 202 mg/g in the empagliflozin group and 250 mg/g in the placebo group, a relative reduction in the empagliflozin group of 19% (95% CI 15–23%). The relative reduction in study average uACR varied substantially between different types of patient, in particular by baseline uACR (Webtable 4). The relative reduction was 5% (6–15%) among patients with baseline uACR <30 mg/g compared with 26% (20–31%) among patients with uACR >300 mg/g.

### Effect of empagliflozin on chronic eGFR slopes

**Figure 2:**
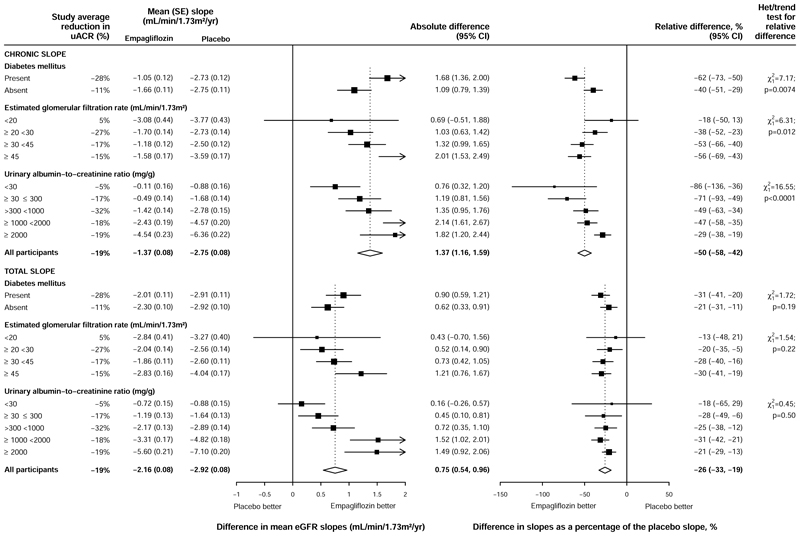
Absolute and relative effects of allocation to empagliflozin on ‘total slopes’ and ‘chronic slopes’, by pre-specified diabetes subgroup, and post-hoc expanded eGFR and uACR subgroups P values for test of heterogeneity between absolute differences in chronic slopes for patients with and without diabetes and tests for trend in absolute differences in chronic slope across eGFR and UACR categories are 0.0085, 0.0013 and <0.0001 respectively. P values for test of heterogeneity between absolute differences in total slopes for patients with and without diabetes and tests for trend in absolute differences in total slope across eGFR and UACR categories are 0.19, 0.023 and <0.0001 respectively.

Overall, allocation to empagliflozin slowed the rate of decline in eGFR from 2 months to final follow-up (the ‘chronic slope’) by 1·37 (95% CI 1·16–1·59) mL/min/1·73m^2^/year, which represented a 50% (42–58%) relative reduction in the mean chronic slope. Larger relative effects were observed among those with diabetes than those without diabetes (62% [50–73%] vs 40% [29–51%] respectively, p for heterogeneity 0.0074, Figure 2). The effect in those with type 1 diabetes was consistent with that seen in those with Type 2 diabetes although the power to detect a difference was low due to the low number of patients with Type 1 diabetes (Webfigure 2). There was some evidence that the relative effects differed across eGFR categories in exploratory analyses splitting out the lowest eGFR category into <20 and ≥20 to <30 mL/min/1·73m^2^ (p for trend 0.01, Figure 2). The treatment effects on the primary composite of kidney disease progression or cardiovascular death across these expanded eGFR categories showed no evidence of effect modification by eGFR however (p for trend 0.81, Webfigure 3).

Smaller absolute differences between empagliflozin and placebo in chronic slopes were observed in the lowest uACR categories, but the mean rate of decline was also substantially lower in these groups. As a result, when comparing the *relative* differences in chronic slope, this trend was reversed with those in the lowest uACR categories having the *largest* relative reduction in the chronic slope (relative reduction 76% [95% CI 32–120%] in those with uACR <30 mg/g compared with 28% [19–38%] in those with uACR ≥2000 mg/g, p for trend 0.0001, Figure 2). The trend seen in the relative difference in chronic slope depending on uACR was similar among participants with and without diabetes (Webfigure 4).

**Figure 3:**
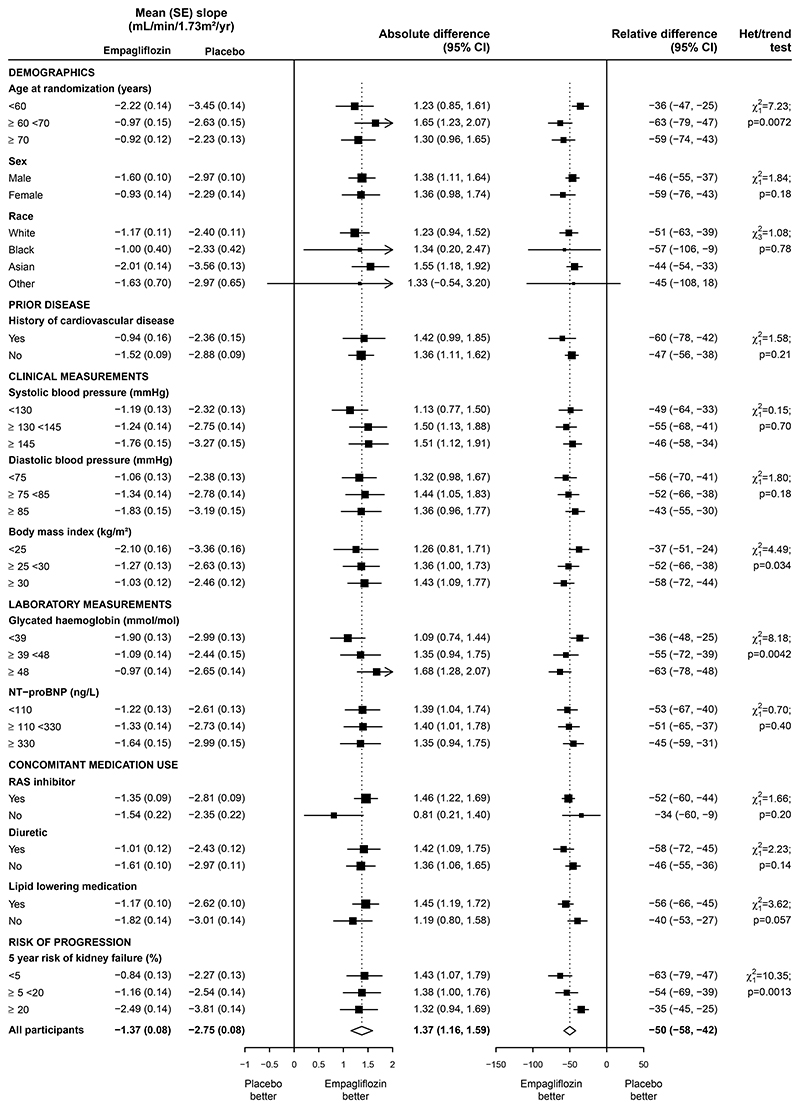
Effect of allocation to empagliflozin on ‘chronic slopes’, by other subgroups

Relative differences in chronic slope were generally similar across other subgroups with statistical evidence for some effect modification by age, glycated haemoglobin and 5-year risk of kidney failure, although clear benefits remained in all subcategories of these subgroups (Figure 3). Differences in the relative effects on chronic slope for the diabetes and uACR subgroups were not explained by their correlation with other key characteristics (as results were essentially unchanged after including interactions with other key subgroups: Webfigure 5). Results were also similar in sensitivity analyses restricted to on-treatment eGFR measurements and using eGFR measurements based on local laboratory creatinine values (Webfigures 6 and 7).

### Effect of empagliflozin on total eGFR slopes

Overall, allocation to empagliflozin slowed the rate of decline in eGFR from baseline to final follow-up by 0.75 (95% CI 0.54-0.96) mL/min/1·73m^2^/year, which represents a 26% (19-33%) relative reduction in the mean total slope (Figure 2). These differences reflect the combinations of the effects on the acute dips in eGFR and the chronic slopes. Relative differences in total slope were similar across all the expanded key subgroups and other subgroups (Webfigure 8).

### Proportion of treatment effect explained by on-study biomarkers

In exploratory analyses, on-study levels of uACR, SBP, DBP and HbA1c explained 41% (95% CI: 23–77%) of the treatment effect on the primary composite of kidney disease progression or cardiovascular death (an attenuation of the hazard ratio from 0.73 [0.63–0.83] to 0.83 [0.72–0.95], and 67% reduction in *χ*^2^ from 20.5 to 6.8; Table 2). uACR alone explained 40% (24–73%) of the treatment effect, while SBP and DBP explained fairly modest proportions (10% [3–23%] and 4% [0–11%] respectively) and HbA1c did not explain any of the treatment effect (0% [-5–4%]; Table 2). Similar patterns were observed for chronic slope, although the proportion of the treatment effect explained by the biomarkers was somewhat lower (26% [19% to 35%]; Webtable 5).

## Discussion

Our analyses show that in this cohort of patients with CKD at risk of progression, allocation to empagliflozin causes a small dip in kidney function of approximately 2 mL/min/1.73m^2^ (or 6%) and then halves the subsequent rate of long-term loss of kidney function. This overall result complements the 29% (95% CI 19–38%) reduction in risk of kidney disease progression when assessed with the categorical composite outcome of end-stage kidney disease, a sustained decrease from baseline in eGFR of at least 40% or to less than 10 mL/min/1.73m^2^, or death from kidney failure. The beneficial effects of empagliflozin on the progression of CKD varied by diabetes status and eGFR, but most prominently by albuminuria, where relative benefits may in fact be larger among participants with lower albuminuria. These findings are consistent with observations in other trials of SGLT2i in CKD, although these trials focussed on patients with diabetes and/or with significant levels of albuminuria.^13,14^ The broad range of patients included in the large EMPA-KIDNEY trial has allowed this to be explored in a more diverse population than those included in other large trials of SGLT2 inhibition in CKD; in particular, EMPA-KIDNEY included participants with eGFR <25 mL/min/1.73m^2^ and with uACR <200 mg/g who were excluded from these previous trials and consequently their analysis of eGFR slopes.

The acute dip in eGFR when empagliflozin was initiated in EMPA-KIDNEY was modest (in all participant subgroups it was on average less than 3 mL/min/1.73m^2^ or <10% of baseline eGFR), and was largely reversible when treatment was discontinued. The acute effect was larger among participants with diabetes compared to those without (on both absolute and relative scales) which may reflect the greater prevalence and degree of hyperfiltration in this group. The acute effect of SGLT2 inhibition on kidney function was recognised early in the development of the class (although not in all studies^23^) and is believed to be due to the acute reduction in intraglomerular pressure caused by afferent arteriolar vasoconstriction stimulated by increased sodium delivery to the macula densa.^24,25^ The associated rapid reduction in albuminuria supports this hypothesis,^26^ and this reduction in intraglomerular pressure is one of the postulated mechanisms of the beneficial effects of SGLT2 inhibition on kidney function.^25^ Our exploratory analyses suggest that the reduction in albuminuria may be the most important measured determinant of the benefits observed in EMPA-KIDNEY, explaining one fifth of the effect on chronic slopes and two fifths of the effect on the primary composite outcome of kidney disease progression, consistent with analyses from other trials in CKD.^27^ These analyses need to be interpreted with some caution as they could be subject to bias due to measurement error and residual mediator-outcome confounding. Whether this association is due to avoidance of direct toxic effects of albumin on tubular function, a reduction in intraglomerular pressure or another unmeasured correlate of urinary albumin is not clear. However, these analyses also suggest that the other mechanisms unrelated to albuminuria, blood pressure or glycaemic control contribute to the benefit of SGTL2 inhibition on kidney function.

Our analyses focussed on chronic slopes. Although effects on total slope correlate strongly with effects on clinical outcomes over short (2-3 year) follow-up periods,^8^ chronic slope is likely to be more informative for longer time horizons. When the magnitude of the acute dip correlates with the relative reduction in chronic slope (which is plausible as they share causal mechanisms such as reduced intraglomerular pressure), this reduces variation between subgroups in total slope when measured over 2-3 years. However, this would not be the case with longer follow-up (see Webfigure 9 for explanatory example). Clinicians seeking to delay or avoid kidney failure would usually consider such longer time horizons for which the chronic slope is most relevant. Furthermore, the limited variation in total slopes between patient subgroups reduces the ability to explore any such differences in treatment effect that may exist between those subgroups. This is demonstrated by the apparent consistency of treatment effect on total slope in EMPA-KIDNEY versus the evidence of effect modification when using chronic slopes.

When comparing chronic slopes, we have reported both the absolute and relative differences but have emphasised the latter. Absolute differences are determined by both the background annual rate of change in eGFR and the relative effect of treatment, so any heterogeneity observed could be due to either of these components. This is demonstrated in the analysis by baseline uACR: the absolute difference in chronic slope among participants with uACR ≥2000 mg/g was 1.84 mL/min/1.73m^2^/yr whereas the background chronic slope among participants with uACR <30 mg/g was 0.99 mL/min/1.73m^2^/yr, so it was impossible for the absolute difference in the latter subgroup to be similar to that observed in the highest uACR subgroup. Indeed the absolute difference in chronic slope was positively associated with baseline uACR; however, the relative difference was inversely associated such that participants with the lowest baseline uACR had the largest relative reduction (76% [32–120%] in those with uACR <30mg/g versus 28% [19–38%] in those with uACR ≥2000 mg/g). There was no strong evidence that this association was importantly modified by the presence or absence of diabetes. Contrary to some international guidelines which only suggest (rather than recommend) using SGLT2 inhibitors in patients without diabetes and without significant albuminuria (uACR <200 mg/g),^28^ these analyses suggest that patients with low albuminuria (with or without diabetes) are likely to gain substantial benefit in terms of preservation of kidney function from SGLT2 inhibition, in addition to the other benefits of reductions in risk of acute kidney injury and cardiovascular disease.^29^ Given the short follow-up in EMPA-KIDNEY (median 2 years) it would be expected that a treatment which causes a 2 mL/min/1.73m^2^ acute dip in eGFR in the subgroup of patients with uACR <30 mg/g (progressing at only 1 mL/min/1.73m^2^/yr) would not demonstrate definitive benefits on the categorical outcome (by contrast with subgroups with higher uACR progressing faster than 2 mL/min/1.73m^2^/yr). These analyses of chronic slope suggest that important benefits would likely emerge with longer treatment (see Webfigure 9 for an illustration).

These analyses are limited by the characteristics of patients included in EMPA-KIDNEY. Few patients with type 1 diabetes mellitus were included, and patients with autosomal dominant polycystic kidney disease or with a kidney transplant were not eligible for the trial. The trial deliberately excluded patients at low risk of CKD progression (i.e., those with eGFR ≥45 mL/min/1.73m^2^ and uACR <200 mg/g), but demonstrated that the relative benefit on chronic slope was inversely proportional to predicted risk of kidney failure. Participants only received study treatment for 2 years on average because the trial was stopped earlier than planned owing to clear evidence of benefit. A further 2 years of off-treatment follow-up is underway to assess the longer-term effects of an average of 2 years of treatment.

In summary, in EMPA-KIDNEY allocation to empagliflozin caused a modest acute dip in eGFR, and then substantially slowed the longer-term progression of CKD. The longer-term benefits varied by diabetes status, eGFR and most prominently uACR (and related characteristics such as predicted risk of kidney failure). Although the trial stopped early because of clear benefits emerging based on results in the highest risk patients, these analyses show that patients at lower risk such as those with lower levels of albuminuria - many of whom in their lifetime would otherwise develop kidney failure - could benefit in terms of preservation of kidney function, in addition to other proven cardiovascular and mortality benefits.^29^ If widely implemented, use of SGLT2i could therefore have a substantial impact on the public health impacts of CKD.

## Supplementary Material

Supplement

## Figures and Tables

**Table 1:** Baseline characteristics by eGFR and uACR

	eGFR (mL/min/1.73m^2^)				uACR (mg/g)			
	<30 (n=2282)	≥30 to <45 (n=2928)	≥45 (n=1399)		<30 (n=1328)	≥30 to ≤300 (n=1864)	>300 (n=3417)	
**Demographics**								
Age at randomization (years)	65 (13)	64 (13)	58 (15)		71 (9)	66 (13)	59 (14)	
Sex								
Men	1533 (67%)	1937 (66%)	947 (68%)		725 (55%)	1268 (68%)	2424 (71%)	
Women	749 (33%)	991 (34%)	452 (32%)		603 (45%)	596 (32%)	993 (29%)	
Race								
White	1440 (63%)	1833 (63%)	586 (42%)		1069 (80%)	1189 (64%)	1601 (47%)	
Black	98 (4%)	119 (4%)	45 (3%)		71 (5%)	89 (5%)	102 (3%)	
Asian	707 (31%)	930 (32%)	756 (54%)		173 (13%)	562 (30%)	1658 (49%)	
Mixed	6 (0%)	13 (0%)	2 (0%)		2 (0%)	6 (0%)	13 (0%)	
Other	31 (1%)	33 (1%)	10 (1%)		13 (1%)	18 (1%)	43 (1%)	
**Prior disease**								
Prior diabetes*	1151 (50%)	1371 (47%)	518 (37%)		647 (49%)	943 (51%)	1450 (42%)	
Prior diabetes type								
Type 1	31 (1%)	28 (1%)	9 (1%)		11 (1%)	20 (1%)	37 (1%)	
Type 2	1106 (48%)	1333 (46%)	497 (36%)		633 (48%)	916 (49%)	1387 (41%)	
Other/unknown	14 (1%)	10 (0%)	12 (1%)		3 (0%)	7 (0%)	26 (1%)	
History of cardiovascular disease§	718 (31%)	828 (28%)	219 (16%)		484 (36%)	579 (31%)	702 (21%)	
**Clinical measurements**								
Systolic blood pressure (mmHg)	137.6 (18.6)	136.0 (18.2)	135.9 (17.8)		130.8 (18.0)	134.3 (17.7)	139.9 (18.0)	
Diastolic blood pressure (mmHg)	76.5 (11.8)	77.9 (11.7)	80.9 (11.6)		73.5 (10.7)	75.8 (11.6)	81.0 (11.5)	
Body mass index (kg/m^2^)	30.1 (6.7)	30.1 (6.9)	28.5 (6.5)		31.5 (7.1)	29.9 (6.6)	29.0 (6.6)	
**Laboratory measurements**								
eGFR (mL/min/1.73m^2^)†								
Mean (SD)	24.6 (3.6)	36.8 (4.2)	59.3 (13.5)		35.1 (8.2)	36.3 (12.8)	38.7 (16.8)	
<30	2282 (100%)	0 (0%)	0 (0%)		386 (29%)	639 (34%)	1257 (37%)	
≥30 to <45	0 (0%)	2928 (100%)	0 (0%)		789 (59%)	896 (48%)	1243 (36%)	
≥45	0 (0%)	0 (0%)	1399 (100%)		153 (12%)	329 (18%)	917 (27%)	
uACR (mg/g)†								
Median (Q1,Q3)	410 (59-1373)	187 (26-781)	515 (214-1199)		7 (6-18)	117 (59-202)	1033 (575-1910)	
<30	386 (17%)	789 (27%)	153 (11%)		1328 (100%)	0 (0%)	0 (0%)	
≥30 to ≤300	639 (28%)	896 (31%)	329 (24%)		0 (0%)	1864 (100%)	0 (0%)	
>300	1257 (55%)	1243 (42%)	917 (66%)		0 (0%)	0 (0%)	3417 (100%)	
NT-proBNP (ng/L)	713 (1681)	470 (1091)	211 (476)		506 (930)	535 (1220)	477 (1384)	
Glycated haemoglobin (mmol/mol)	45.5 (13.7)	45.5 (13.7)	43.1 (13.1)		45.5 (12.1)	45.8 (13.4)	44.3 (14.2)	
Glycated haemoglobin (%)	6.3 (1.3)	6.3 (1.3)	6.1 (1.2)		6.3 (1.1)	6.3 (1.2)	6.2 (1.3)	
**Concomitant medication use**								
RAS inhibitor	1872 (82%)	2487 (85%)	1269 (91%)		1073 (81%)	1545 (83%)	3010 (88%)	
Any diuretic	1151 (50%)	1271 (43%)	393 (28%)		777 (59%)	868 (47%)	1170 (34%)	
Any lipid-lowering medication	1657 (73%)	1955 (67%)	766 (55%)		992 (75%)	1274 (68%)	2112 (62%)	
**Cause of kidney disease**								
Diabetic kidney disease	801 (35%)	901 (31%)	355 (25%)		376 (28%)	623 (33%)	1058 (31%)	
Hypertensive/renovascular disease	533 (23%)	699 (24%)	213 (15%)		469 (35%)	444 (24%)	532 (16%)	
Glomerular disease	452 (20%)	636 (22%)	581 (42%)		66 (5%)	344 (18%)	1259 (37%)	
Other/unknown	496 (22%)	692 (24%)	250 (18%)		417 (31%)	453 (24%)	568 (17%)	

Figures are n (%), mean (SD) or median (Q1, Q3). NT-proBNP=N-terminal pro B-type natriuretic peptide. eGFR=estimated glomerular filtration rate. uACR=urinary albumin-to-creatinine ratio. RAS=renin-angiotensin system.

* Prior diabetes mellitus defined as diabetes at randomization is defined as participant-reported history of diabetes of any type, use of glucose-lowering medication or baseline HbA1c ≥48 mmol/mol at Randomization visit.

§ Defined as self-reported history of myocardial infarction, heart failure, stroke, transient ischemic attack, or peripheral arterial disease.

† Uses central measurement taken at the randomization visit, or more recent local laboratory result before randomization.

**Table 2:** Proportion of treatment effect for primary composite outcome explained by 2 month biomarkers

Biomarkers	Hazard ratio for empagliflozin vs placebo*	Wald χ^2^	% reduction in χ^2^	Proportion of treatment effect explained (95%CI)	
None	0.73 (0.63-0.83)	20.5	0	-	
uACR	0.83 (0.72-0.95)	7.2	65	40% (24% to 73%)	
SBP	0.75 (0.65-0.86)	16.3	21	10% (3% to 23%)	
DBP	0.73 (0.64-0.84)	18.9	8	4% (0% to 11%)	
HbA1c	0.72 (0.63-0.83)	20.7	-1	0% (-5% to 4%)	
uACR, SBP, DBP and HbA1c	0.83 (0.72-0.95)	6.8	67	41% (23% to 77%)	

Analyses restricted to 5465 participants with measurements of uACR, SBP, DBP and HbA1c at 2 months. Participants experiencing an event in the first two months of follow-up were excluded.

* After adjustment for biomarkers at 2 months. All analyses additionally adjusted for baseline variables specified in the minimisation algorithm (age, sex, prior diabetes, eGFR, uACR and region).

## Research In Context

### Evidence before this study

Meta-analysis of the large randomized trials of SGLT2 inhibitors shows that they reduce the risk of kidney disease progression in patients with chronic kidney disease (CKD), as well as acute kidney injury and cardiovascular morbidity and mortality. These analyses were limited by an inability to explore effects in small subgroups or patients whose CKD was only progressing slowly. The EMPA-KIDNEY trial recruited a broad range of patients with CKD, including patients without significant albuminuria. There were relatively few clinical kidney outcomes in this subgroup during 2 years of follow-up, and the results suggested that the proportional benefit of allocation to empagliflozin might be smaller at lower levels of albuminuria. This has led to some uncertainty about the benefits of SGLT2 inhibition in such patients who constitute the majority of CKD patients globally. The use of estimates of the annualised decline in kidney function (“eGFR slopes”) has recently been proposed as an alternative outcome for trials in CKD. It has advantages for exploring the results of large randomized trials because all participants provide information so such analyses are statistically sensitive.

### Added value of this study

These analyses show that empagliflozin caused a modest initial drop in kidney function, followed by a substantial slowing of the rate of progression of CKD in all patients. These ‘chronic’ effects varied by baseline diabetes status, eGFR and uACR in both absolute and relative terms, and these interactions were independent of each other. In particular, the absolute difference in chronic slopes was lower in those with lower baseline uACR, but because this group progressed more slowly than those with higher uACR, this translated to a larger relative difference in chronic slopes in this group.

### Implications of all the available evidence

These results suggest that empagliflozin slows the rate of progression of CKD in a broad range of patients. The findings challenge guidelines which have used albuminuria to stratify their level of recommendation about who should be treated with SGLT2 inhibitors. Patients with lower albuminuria (and consequently at lower risk of progression of CKD) benefit from treatment with SGLT2 inhibition in terms of preservation of kidney function, in addition to reductions in risk of acute kidney injury and cardiovascular disease.
